# College and Hospital Notes

**Published:** 1903-06

**Authors:** 


### COLLEGE AND HOSPITAL NOTES.

"Dr. McMurtry's Infirmary" is the title of a handsome booklet of 16 pages, well printed and beautifully illustrated. It is descriptive of one of the best private hospitals in the country, owned and administered by Professor Lewis S. McMurtry, A.M., M.D., of Louisville, Ky., and which is located at 1912 Sixth Street in that city. The hospital is devoted exclusively to the treatment of diseases peculiar to women, and for cases requiring abdominal surgery; it is a large, double house, three stories in height, standing upon high ground, in the choicest residential district of Louisville, has spacious grounds, modern plumbing, steam heat, bath rooms, open grates, and combines all the elements of a first-class modern home, with the added conveniences necessary to the treatment of the class of patients that it receives. There is nothing lacking that a liberal purse can command or that skilful surgery can supply, to make it an ideal place for patients who seek speedy relief and prompt cure. The hospital has a training school for nurses, and its graduates are among the finest young women in the south, cultivated, kind-hearted and skilled in their profession. The brochure itself is one of the most attractive hospital announcements we have ever seen, and will repay reading by any physician or patient.

---

RESIDENT physicians at the Buffalo hospitals have been appointed as follows: General Hospital, Drs. Edward H. Drozeski, Hyatt Regester, Thew Wright, and Frederick J. Parmenter. Erie County Hospital, Drs. Elliott Bush, Carroll J. Roberts, and Bert J. Bixby. Homeopathic Hospital, Drs. Joseph C. Wallace and Walter L. Williams. The latter are recent graduates in medicine from the Hahnemann Hospital Medical College of Philadelphia.

---

THE Buffalo General Hospital has just issued its forty-fourth annual report. It covers the year 1902, contains 84 octavo pages, is handsomely illustrated, and is as fine a specimen of the printer's art as we have seen in many a day. The total number of patients treated during the year was 2,320, and the total expenditures were $75,925.16. This is a splendid economic showing and indicates not only skilful treatment of the patients committed to its care, but competent financial and business management of the affairs of the hospital. The report is printed by the A. T. Brown press.

